# Non-verbal behaviours as predictors for treatment response in patients with depression or schizophrenia: a systematic review

**DOI:** 10.3389/fpsyt.2025.1643042

**Published:** 2025-12-17

**Authors:** Helena Grant, Elzbieta Vitkauskaite, Arturas Kalniunas, Sofia Pappa

**Affiliations:** 1 West London NHS Trust, London, United Kingdom; 2 Surrey and Borders Partnership NHS Trust, Leatherhead, United Kingdom; 3 Greater Manchester Mental Health NHS Trust, Manchester, United Kingdom; 4 Division of Psychiatry, Imperial College London, London, United Kingdom

**Keywords:** depression, schizophrenia, non-verbal behaviour, precision psychiatry, biomarkers

## Abstract

**Introduction:**

Non-verbal behaviours (NVBs) reveal information about mood and emotions, potentially providing an objective means to measure and monitor early treatment responses. Previous research has examined NVB changes during treatment in patients with depression and psychosis but a systematic evaluation of the evidence is lacking. Furthermore, this review could inform the fast moving field of digital and precision psychiatry due to the use of AI-based technology that could transform the potential of NVBs as reliable biomarkers for treatment response.

**Methods:**

Medline, Embase, PsycINFO and CINAHL were searched in June 2024. Included papers studied adults diagnosed with depression or schizophrenia and measured NVBs at least twice during separate clinical interviews. Outcomes of interest were changes in clinical symptoms and NVBs following treatment initiation. Treatment strategies included hospitalisation, pharmacological, psychological, neuromodulatory, other non-standardised interventions, or a combination of approaches. Two reviewers independently extracted data and assessed risk of bias. The protocol for the review was registered on PROSPERO.

**Results:**

20 papers were identified; 15 on depression, two on schizophrenia and three evaluating both conditions separately. Methodological variations across studies made comparisons challenging. NVBs consistently associated with improvement in depression symptoms included: increased smiling, facial expressivity, and amplified head and body movements. Results across studies were more consistent when considering general categories of behaviour, versus discrete facial behaviours. No commonalities were observed in NVB changes over time for patients with schizophrenia.

**Discussion:**

The existing evidence is presently insufficient to establish distinct behavioural profiles for clinical improvement depression or schizophrenia. Despite implicit challenges, there is considerable future scope in the evaluation of NVBs as predictors of clinical outcomes or change.

**Systematic Review Registration:**

https://www.crd.york.ac.uk/PROSPERO, identifier CRD42022368599.

## Introduction

Non-verbal behaviour (NVB) is defined as any movement or position (including distance and proximity) of the face and/or body (1). Typical NVBs include facial expressions, gestures, eye contact and posture. NVBs are a rich source of information less influenced by conscious control that provides additional insight into a person’s internal state compared to information conveyed through speech alone (2).

The study of NVB in psychiatric conditions began in the 1960s (3). However, ethological methods applied to the diagnosis and treatment of mental illness have been only used occasionally in the years since, and the potential of NVBs as a source of clinical information remains largely underutilised.

The current approach to diagnosing psychiatric illness and assessing treatment response often relies on unstructured clinical consultations or the use of rating scales and questionnaires. Although some commonly used rating scales can assess observable physical behaviours, there is potential for integrating automated methods or more objective means of analysis to enhance the assessment process. For example, the Bush-Francis Catatonia Rating Scale is a valuable tool for the diagnosis and monitoring of catatonia and recent research highlights the utility of measuring catatonia motor behaviour with objective instrumentation, suggesting a promising avenue for future advancements in the field (4).

Generally, there appears to be a pressing need for objective measures that can accurately determine clinical changes in response to treatment, addressing limitations and enhancing the role of traditional rating scales (5, 6).

The predictive value and importance of closely monitoring treatment responses in psychiatric illnesses, particularly in the early stages, have been highlighted over recent years. A meta-analysis found that early improvement in depression treatment was linked to better outcomes in 15 out of 16 studies (7). In addition, a review of four randomised controlled trials involving 1–627 patients with schizophrenia found that 53% of patients stop treatment early, with poor symptomatic response being the primary reason (8). NVBs are an example of potential behavioural biomarkers (i.e. objective indicators derived from behaviour) that can be quantified to measure treatment response that may be superior to the use of rating scales, providing earlier indications of therapeutic response and leading to improved patient outcomes (9, 10).

The utility of NVBs in psychiatry is derived from the bidirectional relationship between internal emotions and outwards expressions conveyed through facial expressions or movement. Sensory feedback from facial and bodily movements can contribute to internal emotional experiences (2, 11, 12), and the theory of embodied emotion describes how emotional expression, perception and processing are related to internal emotion and arousal (13). A more granular understanding of the emotions and symptoms underlying movement and behaviour could give further clinical relevance to the use of behavioural metrics for measuring treatment response, rather than just quantifying the change in NVB in isolation.

Reduced reactivity and aversive behaviours are consistent with evolutionary theories of depression, such as the social withdrawal hypothesis, and highlight the manifestation of psychomotor retardation (14–16). Thus, a reduction in psychomotor retardation, withdrawal behaviours and a subsequent increase in movement behaviours could be linked to reduced symptom severity and clinical improvement in patients with depression.

The relationship between movement and emotion in schizophrenia is more challenging to discern due to the potential confounding effect of antipsychotic medications on movement. However, research has shown that greater levels of pro-social behaviours are conducive to reduced symptom severity (17).

Additionally, the relationship between NVB and clinical outcomes, particularly in the therapeutic setting, could be mediated through the synergy of NVBs and the therapeutic alliance between patient and clinician. Research has found that patients with schizophrenia who exhibited more social behaviours towards their psychiatrist reported greater satisfaction with communication and a better therapeutic relationship, contributing to more favourable outcomes (17). In patients with depression, greater behavioural synchrony between patients and interviewers has been associated with reduced severity of depression over the course of the illness (18).

NVBs can be quantified using manual or automatic coding methods. Manual methods employ trained raters to watch videotaped subjects frame by frame and record each behaviour that occurs, using frameworks such as the Facial Action Coding System (FACS) developed by Ekman and Friesen (19). FACS objectively codes facial behaviours based on facial anatomy and divides them into Action Units (AUs which represent small visually discriminable changes in facial movements, making it an accurate and powerful measure (20, 21). Automated methods are being increasingly employed to analyse NVBs and increase efficiencies in the labour-intensive coding process. One study comparing manual and automated coding of nonverbal synchrony found that manual coding is comparable to automated methods in terms of accuracy, yet the process is greatly more efficient. The authors illustrated that the manual coding of 110 subjects, including time taken to develop the coding system, train and establish reliability among coders took approximately 12 months, whereas automated coding of the same participants took 2 weeks (22). Moreover, using AI-based and automated methods provide a more objective lens for interpreting findings that are not influenced by the assessors’ emotions or other variable factors. AI models support feature extraction from complex facial data, reducing the need for manual feature extraction and analysis (23). An application of this is the use of convolutional neural networks and machine learning models to study the early detection of depression, for example through comparing data from depressed participants and healthy controls to automatically estimate the likelihood of depression based on differences in facial features (24).

The potential of NVB as an objective biomarker for disorder progression has been highlighted in recent technology-enabled studies which have demonstrated new methods of objectively measuring symptoms. For example, using remote measurements of facial expressions, movement, voice and speech, sleep, and general activity to monitor symptoms, responses to treatment and risk of relapse in psychiatric populations (25, 26).

The field of technology that can analyse behaviour is expanding rapidly, yet before more advanced ways of monitoring responses to treatment can become widely used, it is beneficial to understand the existing knowledge base in relation to the potential use of NVBs as markers of response to treatment in serious mental illness. To date, there has been no systematic evaluation of the literature on how NVBs change in response to treatment and only a few narrative reviews on the wider topic of NVBs are presently available (2, 9, 17, 27, 28). This review aims to systematically evaluate the existing literature, and compare and synthesise findings across studies to identify NVBs that could be used to assess improvement or deterioration in response to treatment. The review will also assess the quality and methodologies of existing studies and make suggestions for future research.

## Methods

This review was guided and reported in accordance with PRISMA guidelines (29). The review protocol is registered in PROSPERO and is available online (reg: CRD42022368599).

### Eligibility criteria

The inclusion and exclusion criteria are presented in **Table 1**.

**Table 1:** The eligibility criteria used for paper selection.

Study characteristics	Inclusion	Exclusion
Type of record	• Observational, longitudinal studies • English language only	• Conference papers • Book chapters • Presentations • Review articles • Case studies
Population	• Adults aged 18 or over • Patients with a diagnosis of schizophrenia or depression (unipolar or bipolar) • Receiving inpatient or outpatient care	• Patients under the age of 18 • People with learning difficulties or cognitive impairment
Intervention	• Any treatment (psychological, pharmacological, hospitalisation) over any length of time	• Not receiving any treatment/intervention
Comparison	• Comparing non-verbal behaviours before, during or after treatment at multiple time points	
Outcome	• Improvement or worsening of symptoms • Relapse or remission • Changes in non-verbal behaviour	
Study design	• Any method of behavioural analysis • Observing behaviour during a clinical interview at multiple time points during treatment	• Comparing patients with healthy controls • Looking at behaviours at one time point only • Behaviours not recorded during a clinical interview

#### Population

The populations of interest were adult inpatients or outpatients with a diagnosis of a serious mental illness namely depression or schizophrenia. Other psychiatric disorders such as anxiety disorders, eating disorders, obsessive compulsive disorder, personality disorders were not in the scope of this review. The literature on NVBs in these conditions is limited, and treatment strategies are more varied and heterogeneous, which would not enable results to be synthesised to illustrate a profile of NVBs in these patients.

#### Intervention

As the literature in this area is limited, limiting the search to specific interventions could have yielded too few studies for a meaningful synthesis of the evidence. Therefore, only studies where patients did not receive any treatment or intervention were excluded.

#### Comparator

Included studies compared NVBs at multiple time points (at least twice) throughout the course of treatment to study how NVB changes alongside clinical improvement, stabilisation or deterioration. Only studies assessing NVB during a clinical interview were selected as this is the most standardised means for measuring NVB across studies, as opposed to image viewing tasks or naturalistic observation which display a greater degree of variation across studies. The search aimed to identify cohort studies where the same group of patients were observed over time to study how NVBs change over time rather than identifying how NVBs differ in patients versus healthy controls.

Any study that analysed changes in NVB and clinical change over time was included. The relationship could be assessed using any methodology or statistical test - for example, simple group comparisons to assess treatment-induced changes, or more advanced analyses to explore if NVBs could predict clinical change. Due to the already small evidence base, studies were not excluded based on analysis approach.

#### Outcome

The outcome measures studied were clinical improvement or deterioration over the duration of the study, measured using any method such as clinical rating scales or clinical interviews (30). Changes in NVBs could also be assessed using method. No exclusions were placed on the approach used to measure outcomes, again to avoid narrowing the scope too extensively. Whilst NVBs can also encompass speaking behaviours such as speech rate, pitch, tone, volume, rhythm, as well as physical movements, these were not in the scope of this particular review.

### Information sources

The databases searched were Medline, Embase, PsycINFO and CINAHL. The search was conducted in May 2022 and updated in June 2024 to include new research prior to publication. Reference lists of included studies were screened to identify additional papers.

### Search strategy

The final search strategy is presented in the **Supplementary Materials**. No limits were placed on the date or location of publication. An English language filter was applied to the search. The search strategy was created through an iterative process where HG, EV, SP and AK all inputted.

### Selection process

Results from the search were imported into Rayyan for screening (31). HG and EV independently screened the title and abstract of all papers. The interrater reliability at this stage was 0.7 (Cohen’s Kappa). Once all abstracts were screened, HG and EV undertook full-text screening of all remaining papers. HG and EV discussed disagreements in the records that were included or excluded, and in cases where a decision could not be made, SP and AK were consulted.

### Data extraction

Key outcomes data collected were author, year of publication, country, study design and setting, assessment tool for NVB and clinical change, statistical tests and method of aggregation, type and length of intervention and outcomes and main results. Data from each study were extracted into an Excel spreadsheet. Three studies were subject to an initial data extraction by HG and EV and compared to confirm that the result was satisfactorily similar. The interrater agreement for the data extraction process was 83%. HG and EV discussed any discrepancies before proceeding with the data extraction for the remaining studies independently. The full data extraction form is presented in the **Supplementary Materials**.

### Risk of bias assessment

The CASP cohort study checklist was used to assess the risk of bias in each included paper (32). Three studies were subject to an initial quality assessment pilot by HG and EV and were compared to confirm that the result was similar. HG and EV extracted data from the remaining papers independently. Disagreements were to be resolved by separate discussions with SP and AK.

### Data synthesis

Due to the heterogeneity of methods used to measure and record NVB, formal synthesis and meta-analysis of results were not possible. Results were subject to narrative synthesis by summarising the main outcomes of each paper and determining which changes in NVB were significantly associated with clinical improvement or deterioration. We compared the behavioural profiles for depression and schizophrenia, and whether these differed across intervention types. We also explored the overall quality and strengths and weaknesses of the evidence and highlighted key knowledge gaps and avenues for future research.

## Results

### Study properties

**Figure 1:**
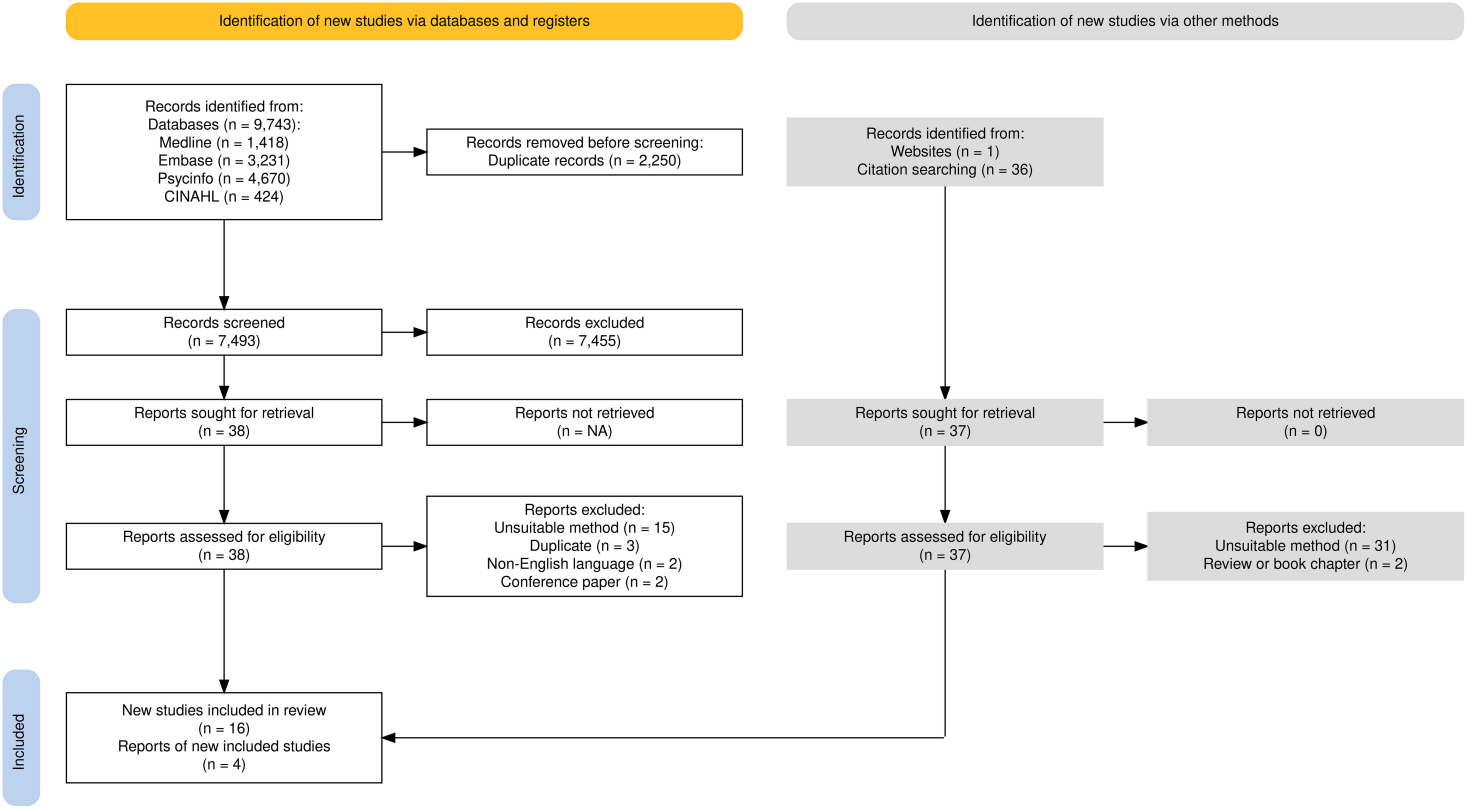
PRISMA flow diagram (33).

We identified 9–743 publications from the searching stage and screened 7–493 titles and abstracts after removal of duplicates. Following abstract screening, 75 studies underwent full text screening, and 20 studies met the final eligibility criteria for inclusion (**Figure 1**). **Table 2** summarises the properties of included studies. 15 studies observed patients with depression, two studies observed patients with schizophrenia and three studies observed separate groups of patients with depression and patients with schizophrenia within the same study.

**Table 2:** Properties of all included studies.

Authors	Location	Diagnosis	Setting	Number of participants	Intervention	Study length	Method of outcome assessment	Method of NVB analysis	Nature of interview	Statistical analysis	Key results
Bhatia et al., 2019 (34)	USA/ Australia	Major depressive disorder	Outpatient	47	Not specified	21 weeks	HRSD	Automated analysis of videotaped interview to determine interpersonal synchrony in head movements between the patient and therapist	Structured interview with therapist using HRSD	Hierachichal linear model comparing nonverbal synchrony between patients and therapists over the course of treatment as depression severity scores changed	Head movement synchrony did not change over the course of treatment, results were not statistically significant
Bouhuys & Albersnagel 1992 (35)	The Netherlands	Major depressive disorder or bipolar depression	Inpatient	31	Various (tricyclics, antipsychotics nonpharmacological therapies not specified), intervention given for up to 87 days or hospital discharge	Up to 10 weeks	HRSD	Manual analysis of videotaped interview Coded behavioural categories included vocalisation, looking, head movements, hand movements, leg movements which were grouped into different factor categories (restlessness 1 [leg movements and light body touching during speaking and listening], restlessness 2 [object touching], active listening [intensive body touching, head movements] speech, and eagerness [yes nodding and no shaking]	Structured interview with psychiatrist using HRSD	Repeated measures MANOVA comparing NVB changes over the course of treatment between improved and non-improved patients	Non-improved patients displayed more speaking effort (p=0.038), restlessness 1 (p=0.076), and less active listening (p=0.053) than improved patients. Over time, improved patients showed increased restlessness 1 (p=0.009), speech (p<0.001), eagerness and speaking effort (p<0.001). Restlessness 2 (p=0.008) and active listening decreased over time (p=0.026)
Bouhuys et al., 1986 (36)	The Netherlands	Major depressive disorder	Inpatient	31	Various (tricyclics, antipsychotics, nonpharmacological therapies not specified, combination) given for up to 10 weeks	10 weeks	HRSD	Manual analysis of videotaped interview Coded behavioural factors included sound production, looking, head movements, encouragement behaviours, hand movements and leg movements	Structured interview with psychiatrist using HRSD	Mann-Whitney U test comparing NVBs in improved and non-improved patients after treatment	Improved patients showed fewer looking behaviours compared to non-improved patients (p<0.02) Light body touching was significantly correlated with the degree of improvement (p<0.05) Significant positive correlation between improvement and head movements during patient listening, and during the pause before their speaking turn
Bouhuys et al., 1987 (37)	The Netherlands	Major depressive disorder	Inpatient	60	Group 1: clomipramine, clomipramine and sleep deprivation, combination of placebo and sleep deprivation (n=29), given for 1–2 weeks Group 2: Various (tricyclics, antipsychotics, nonpharmacological therapies not specified, combination) (n=31) given for up to 10 weeks	Up to 10 weeks	HRSD	Manual analysis of videotaped interview Coded behavioural factors included sound production, looking, hand movements, head movements	Structured interview with psychiatrist using HRSD	Pearson correlations to assess correlation between looking behaviours and HRSD score Mann-Whitney U test comparing NVBs in improved versus non-improved patients after treatment	Over 2 weeks: Improved patients showed higher amounts of looking than those who did not improve (p<0.05) and looking was significantly correlated with the degree of subsequent improvement (p<0.01) Patients with lower levels of object touching hand movements at the start of their speaking turn have more chance of improving than patients showing high amounts Improved patients showed higher amounts of gestures than non-improved Improved patients showed more yes nodding than non-improved Over 10 weeks: Improved patients showed larger proportions of intensive body touching (p<0.02) Improved patients showed less gesturing than non-improved patients (p<0.05) Frequency of yes nodding was larger in non-improved than improved patients (p<0.05), but improved patients showed a greater duration of head movements (p<0.05)
Fiquer et al., 2013 (38)	Brazil	Major depressive disorder	Outpatient	40	Transcranial direct current stimulation, given daily for 2 weeks	2 weeks	HRSD, BDI, BAS, life satisfaction score	Manual analysis of videotaped interview using ethogram with categories including posture, face movements and expressions, hand movements, head posture and movement, looking, vocalisation	Clinical interview where patient describes current mood state	Repeated measures ANOVA comparing changes in depression scores and NVBs before and after treatment Mixed model analysis evaluating if NVB changes before and after treatment was associated with changes in depression scores, and if behaviours at baseline predicted depression scores after treatment	After treatment, patients showed reduction in behaviours associated with negative emotion and low energy (lips down, frowning, crying, head down), and an increase in behaviours related to interpersonal interest (yes nodding and eye contact) (p<0.05)
Fiquer et al., 2017 (39)	Brazil/The Netherlands	Major depressive disorder	Outpatient	25	Sertraline 25-200mg per day for 8 weeks	8 weeks	HRSD	Manual analysis Coded behavioural features included gaze, general head movements and gestures during speaking; gaze during listening, the interviewer's verbal backchannel and yes nodding during listening	Structured interview with psychiatrist using HRSD	Repeated measures ANOVA and Pearson correlations to assess changes in NVB over time and association with symptom severity	Improvers showed no significant change in behaviour. Patients who didn’t respond to treatment showed more speaking effort behaviours after 8 weeks (p=0.04)
Fisch et al., 1983 (40)	Switzerland	Major depressive disorder	Inpatient	13	Various during hospitalisation (specifics not reported), length of intervention not reported	Between 25–77 days	10-point analog scale ranging from "very depressed" (10) to "fully recovered" (1).	Manual analysis of videotaped interview Used time-series notation method, which coded the dimension and scale of head movements, trunk movements, shoulders, upper arms, hands, upper legs, feet and position on chair	Interview with psychiatrist discussing patient's health problems	Paired sample t-tests and Pearsons correlation to compare NVBs before and after treatment	Global time spent in motion increased over time (p<0.01) Complexity of body movement (the degree to which various parts of the body are simultaneously involved in movement) increased over time (p<0.01) Recovered patients showed significantly more behavioural dimensions than in the depressed state (p<0.01)
Fisher et al., 2023 (41)	Israel	Major depressive disorder	Outpatient	86	16 sessions of manualised psychotherapy	16 weeks	PANAS	Automated analysis using AFFDEX classifier which is based on FACS Measured valence which indicates whether the emotional state is positive or negative	Recorded therapy session	Regression models to assess changes in NVB during treatment and if emotional valence can predict treatment scores	Increase in valence was positively associated with greater perceived positive emotions and a decrease in negative emotions over the course of therapy Valence was shown to be significantly predictive of change in HRSD (p=0.018)
Gaebel & Wölwer 2004 (42)	Germany	Schizophrenia and major depressive disorder	Inpatient	92 Acute schizophrenia: 33 Partly remitted schizophrenia: 36 Acute MDD: 23 Nonpatient controls: 21	Antipsychotics or antidepressant, length of intervention not reported	3 months	BPRS, SANS, HRSD, EPS	Manual analysis using FACS to code the intensity and repertoire of action units	Discussion of present state and past good and bad experiences to recall range of emotions	Repeated measures MANOVA to compare NVBs before and after treatment	Both groups saw a reduction in facial activity (p<0.001) and repertoire (p=0.001) over 4 weeks of treatment which stabilised over the subsequent 3-month period Flattened facial activity resulted from reduced upper face AUs (AU1, AU2, AU4) and AUs shown in relation to positive emotion or communicative functions (AU6, AU12)
Girard et al., 2013 (43)	USA	Major depressive disorder	Unknown	18	Interpersonal psychotherapy or selective serotonin reuptake inhibitor (specific medication and length of intervention not specified)	Not reported	HRSD	Manual analysis using FACS and automated analysis of videotaped interview	Structured interview using HRSD	Paired sample t-tests comparing NVBs before and after treatment	Manual analysis: Improved patients showed Increased AU12 and decreased AU14 (p<0.05) and increased AU15 (p<0.1) Automatic analysis: Increased AU12 (p<0.1) and AU15 (p<0.05) and decreased AU14 (p<0.05) as patients improved Both manual and automatic coding showed a significant reduction in the proportion of smiling frames
Girard et al., 2014 (16)	USA	Major depressive disorder	Unknown	19	Interpersonal psychotherapy or selective serotonin reuptake inhibitor (specific medication and length of intervention not specified)	21 weeks	HRSD	Manual analysis using FACS and automated analysis using facial landmark tracking and head pose tracking of videotaped interview Behaviour categories included AUs 12, 14, 15, and 24 and head motion	Structured interview using HRSD	Paired t-test comparing NVBs in patients before and after recovery	Manual analysis: As patients improved, AU12, AU14 and AU15 significantly increased (p<0.05) Automated analysis: As patients improved, AU14 significantly decreased and AU15 significantly increased (p<0.05) Head motion increased in vertical amplitude (p<0.05) and vertical velocity, horizontal amplitude and horizontal velocity (p<0.01)
Jiang et al., 2021 (44)	USA	Major depressive disorder	Inpatient	12	6 months of deep brain stimulation	8 months	HRSD	Automated analysis using convolutional neural network-based automated facial expression recognition	Psychiatric assessment interview. Interviews were unstructured to include spontaneous conversation and unscripted response to typical psychiatric assessment questions	Mann-Whitney U test comparing NVBs in improved and non-improved patients after treatment	Patients who did not remit show significantly lower expressions associated with disgust, happiness (p<0.005) and surprise (p<0.02) and significantly higher sadness (p=0.04) and anger (p<0.005)
Jones & Pansa 1979 (30)	Australia	Depression and Schizophrenia	Inpatient	25 Schizophrenia: 12 Depression: 13	Various during hospitalisation (specifics and length of intervention not reported)	4 weeks	HRSD, BPRS, HAS	Manual analysis using ethogram of facial and looking behaviours, including head movements, body contact, looking behaviour, smiling, eyebrow movements	Interview asked about social and demographic data such as name, age and occupation, then asked about clinical features that led to admissions	Paired sample t-tests comparing NVBs before and after treatment	Increase in small head movements (p<0.05) and body contact (p<0.01) over time in patients with schizophrenia, increased smiling over time in patients with depression (p<0.05)
Liu et al., 2022 (45)	China	Major depressive disorder	Inpatient and Outpatient	164	7 sessions of systemic psychotherapy	7 sessions - length not specified	Self-rating depression scale Self rating anxiety scale, HRSD	Automated analysis of BDD (behavioural depression degree) which encompassed expression entropy (EE) (high EE = lively expression, low EE =simple expression) using convolutional neural network-based automated facial expression recognition	Recording of psychotherapeutic interviews	Pearson correlation to assess changes in NVB over treatment	Level of BDD reduced as clinical improvement occurred
Mackintosh et al., 1983 (46)	England	Depression and Schizophrenia	Inpatient	23 Endogenous depression with psychomotor retardation: 9 Endogenous depression without psychomotor retardations: 7 Depressive neurosis: 7 Schizophrenic with depressive symptoms: 5	Tranquilisers (major or minor) or tricyclic antidepressant for 6 weeks	6 weeks	HRSD	Manual analysis where frequency of blinking was determined using a push-button counter	Present state examination interview	ANOVA comparing changes in blink rate during treatment	Depressed participants showed a significant reduction in blink rate over treatment (P<0.001)
McCall et al., 2020 (47)	USA	Major depressive disorder	Outpatient	12	6 months of deep brain stimulation	6 months	HRSD and BDI	Manual coding using ethogram developed for study which included eye and upper face, head movements, mouth movements, expressions, gestures, posture and speech. Individual behaviours were grouped into factors for the analysis	Structured interview with psychiatrist using HRSD and BDI	Factor analysis to group NVBs into factors, and two-way repeated measures ANOVA to assess changes in factors over time. Mixed effects modelling assessed the relationship between BDI score and NVB frequency	After treatment, patients showed more reactive, engaging and fidgeting behaviours, and less behaviours associated with disengagement (p=0.032)
Meya & Renfordt 1986 (48)	Germany	Paranoid schizophrenia ^I^	Inpatient	24	28 days of haloperidol or perazine	4 weeks	AMP score	Manual analysis where duration and total frequency of eye contacts and the proportion of time during which eye contact occurred was measured	Structured interview using AMPS system	Wilcoxon Rank and Mann Whitney U tests to compare changes in NVB during treatment, Spearmans Rank correlations to assess relationship between NVBs and symptom scores	As symptoms reduced, a significant negative correlation was observed with the time spent in eye contact at day 7 (p<0.04), day 14 (p<0.03) and day 28 (p<0.02)
Paulick et al., 2018 (49)	Germany	Depression	Outpatient	68	Cognitive behavioural therapy, 13–83 sessions	Up to 83 sessions - length not specified	SCID-I, BSI	Automated motion energy analysis to analyse nonverbal synchrony	Videotaped CBT session	Multilevel modelling to assess association between nonverbal synchrony and movement during treatment	Lower levels of nonverbal synchrony at the beginning of therapy in patients with depression were predictive of greater symptom reduction (p=.02). Patient movement quantity significantly reduced at the end of treatment (p<0.05)
Schneider et al., 1992 (50)	Germany	Schizophrenia or schizophreniform disorder	Inpatient	16	Group 1: Unmedicated at the first session, then given antipsychotics immediately after (n=8) Group 2: Medicated at first session and continued taking medication (n=8)	3 weeks	GAS, BPRS, SANS	Automated analysis using small round foils attached to different regions of the patients face, facial action points corresponding to FACS AUs were analysed by a computer	The patient was asked questions about their general well-being, about the illness, and about how the individual was coping with his present situation.	Paired sample t-tests comparing NVBs before and after treatment	A significant reduction in facial actions over time occurred in group 1 who were unmedicated at T1 (p<0.037). Facial actions in group 2 who were medicated at T1 showed no significant change. The nonmedicated patients showed a significant reduction in AU repertoire/variability of facial expressions at T2 in comparison to T1 (p=.034). AU1 and AU4 (p=0.015) and AUs of the lower face (p=0.022) reduced in frequency over time in group 1 Facial action of patients in group 2 remained unchanged over time
Ulrich & Harms 1985 (51)	Germany	Depression	Inpatient	47	Antidepressant medication (specifics and length of intervention not reported)	3 weeks	Not measured	Manual analysis of categories including retardation, agitation, reduced eye movements, reduced facial expression, constricted posture, postural restlessness, low voice and diminished prosody	Structured interview using AMPS system	Factor analysis comparing NVBs before and after treatment	When comparing factors before and after treatment, there was less postural restlessness and discrete body touching, more facial expressiveness and less low voice, reduced continuous undirected hand activity and more continuous directed hand activity Patients showed an overall decrease in syndrome complexity, no significance values or numbers given

AMPS, anxiety, mood psychosis, substance use; AU, action unit; BAS, Brief Affectivity Scale; BDI, Beck Depression Inventory; BPRS; Brief Psychiatric Rating Scale; CBT, cognitive behavioural therapy; EPS, Extrapyramidal Side Effects Rating Scale; GAS, Global Assessment Scale; FACS, Facial Action Coding System; HAS, Hamilton Anxiety Scale; HRSD, Hamilton Rating Scale for Depression; NVB, nonverbal behaviour; PANAS, Positive and Negative Affect Scale; SANS; Scale for the Assessment of Negative Symptoms; SCID-1, Structured Clinical Interview for DSM.

^I^Paranoid schizophrenia is defined within the study as aligning with the corresponding ICD code (52). In short, in this subtype of schizophrenia, delusions and hallucinations are common but thought disorder, disorganised behaviour, and affective flattening are not prominent.

Study sample sizes ranged from 12–164 participants, with the average being 43 participants. Interventions included pharmacological intervention, psychological intervention, neuromodulatory intervention, and a variety of non-standardised interventions including hospitalisation, pharmacological and psychological interventions. Interventions ranged in length from 2–32 weeks, with the average length being 12 weeks. Most studies (14/20) assessed patient outcomes using The Hamilton Rating Scale for Depression (HRSD).

Regarding the analysis of NVBs, 12/20 studies used manual analysis, 6/20 used automated analysis, and 2 studies compared both automated and manual methods. NVB was assessed most frequently during a videotaped clinical interview based on the administration of the HRSD. Other assessment settings included videotaped psychotherapy sessions or less structured mental state examinations.

Most studies (15/20) used group comparison tests or correlations to compare NVBs before and after treatment, showing treatment induced changes in NVBs. Five studies used multilevel modelling or regression approaches, and two studies extended the analysis to make predictive inferences about how NVBs may predict clinical change. Fiquer et al. (2013) (38) found that adaptive gestures (self-touching and object touching) at baseline predicted higher negative affect scores after treatment (p=0.04). Fisher et al. (41) found that an increase in emotional valence during the therapy session predicted later reductions in depression symptom scores (p=0.018).

Heterogeneity across studies made synthesis challenging, mostly due to the wide variety of behavioural categorisation and measurement approaches used. For example, some studies focused on measuring specific facial AUs and discrete movements, whereas others grouped behaviours into broader categories and ascribed emotional states (such as happiness, anger and restlessness) to patterns of NVBs behaviours. This led us to focus our synthesis on broader categories of behaviour. A wide variety of methods to measure NVBs and lack of standardised procedure also impaired comparisons. Some studies measured NVB continuously throughout the clinical interview, others for just a short portion of the interview, and the features of NVB that were recorded varied across studies (e.g. duration, frequency and intensity). It was also challenging to determine if automated methods led to more consistent results due to the still broad range of approaches used to measure and categorise behaviour among this subset of studies. Among the studies employing automated coding methods, NVBs measured included nonverbal synchrony between the patient and interviewer (34, 49), emotional valence (41), changes in AUs (16, 43, 50), changes in behaviours associated with specific emotions (44), behavioural depression degree (45). This emphasises the need for more standardised measurement approaches to strengthen conclusions.

### Overview of behavioural changes

Definitions of key NVBs discussed in this section are presented in **Table 3**.

**Table 3:** Behaviour definitions.

Non-verbal behaviour	Description
Blink rate	Frequency of blinks within a defined time period (46)
Body contact/touching	Movement by which the subject makes contact with their body such as touching the face or manipulating the hands (35)
Body movement	Any increase in the quantity and range of movements, e.g. increased mobility and complexity of behaviours and movements (40)
Disengagement behaviours	Slow speaking, pausing, looking down, reduced looking (47)
Emotional valence and expressivity	Change in facial expressions corresponding to specific emotions (e.g. angry, happy, neutral, sad) (44) or emotional valence (positive or negative) (41)
Expression entropy	High expression entropy is defined as more lively expressions, low expression entropy is defined as more simple expressions (45)
Facial expressivity	Number of identified action units occurring per minute (intensity of expression) or number of different AUs shown at least twice during the interview (repertoire) (42)
Gesturing	Hand or arm movement use to support speech (47)
Head movement	Any movement of the head, including nodding, shaking, head tilted to the side, up or down (47)
Mouth movements/smiling	Any movement of the mouth – corresponding to action units of the lower face (FACS). Smiling corresponds to AU12 and contraction of the zygomatic major muscle (16)
Nonverbal synchrony	Coordinated movements between patient and interviewer (34)
Speaking effort	Looking, gesticulating and head movements during speech (35)

### Facial behaviours

The synthesis of results firstly focused on the analysis of behaviours relating to smaller, discrete parts of the face (e.g., eye and eyebrow movements, mouth movements) and the relationship between discrete behaviours and responses to treatment. Results were highly variable, and the only consistently occurring facial behaviour associated with symptomatic improvement in patients with depression was increased mouth movements/smiling, observed in 4/18 studies (16, 30, 43, 47). Only two studies assessed facial behaviours in patients with schizophrenia, therefore a robust conclusion cannot be drawn, although both studies both observed a significant reduction in facial activity and expressiveness over time (42, 50).

### General NVBs

Due to the lack of consistency across specific facial behaviours, we focused our analysis on more general behaviours such as body movements, gesturing, emotional expressivity, and emotional valence. Studies seemed to shift from more specific methods of analysing NVB in older studies (e.g., using FACS or ethograms comprised of specific muscle movements) to a more global and generalised approach of assessing the nature of NVB changes in studies published from 2021 onwards. A more generalised approach may enable inter-individual differences in specific facial movements and differences in methods of measuring NVB to be accounted for. 16/20 studies conducted a more global assessment of behaviours (as opposed to assessing facial AUs only or blinking/eye movements), 14 studies included patients with depression in the sample, and 2 included both patients with depression and patients with schizophrenia in the sample.

### Depression

Generally, as clinical improvement occurred over time, patients with depression showed increasing levels of social and affiliative behaviours, including head movements (5/14 studies) (16, 35–37, 47), gesturing (2/14 studies) (37, 47), body contact/touching (35–37) (3/14 studies), facial expressivity and positive emotional valence (6/14 studies) (38, 41, 44, 45, 47, 51), movement (including restlessness and fidgeting) (3/14 studies) (35, 40, 47). Such behaviours portray an image of the patient transitioning from behaviours associated with social withdrawal and diminished emotions to more active and engaged as treatment progressed. Although increased levels of restlessness and fidgeting could also be interpreted as occurring due to discomfort. Hence, despite the differing definitions of body movement and restlessness, many studies did not differentiate between the two behaviours nor acknowledge that restlessness and fidgeting could be associated with negative valence.

Conflicting results were reported by Bouhuys and Albersnagel (1992) (35) and Fiquer et al. (2017) (39) who found that patients who did not improve showed more speaking-effort behaviours which contrasts with the theory of pro-social behaviours being associated with improvement. In addition, Gaebel and Wölwer (42) (2004) reported that as symptoms improved, facial expressivity and emotional repertoire reduced.

### Schizophrenia

No consistently occurring behaviours were observed across the two studies which assessed more general behaviours in patients with schizophrenia (30, 42).

### Effect of intervention type on general NVBs

7 of the 16 studies that measured generalised behaviours assessed the association between NVB and pharmacological intervention (16, 35–37, 39, 42, 51). This subset of studies corroborated with some of the overall findings which showed an increase in body touching (35), gesturing (51), nodding and head movements (16, 35–37), and a general increase in expressivity and AUs (16, 51) as patients received pharmacological intervention. One conflicting result in this subset of studies was that of facial expressivity, Ulrich and Harms (1985) found increased facial expressivity as symptoms improved whereas Gaebel and Wölwer (2004) showed reduced facial expressivity after 3 and 4 weeks of antidepressant treatment respectively.

3 studies measured the effect of neuromodulatory treatments (2 deep brain stimulation (44, 47), 1 transcranial direct current stimulation (38)) and found that patients showed increased nodding and eye contact after 2 weeks of transcranial direct current stimulation and increased facial expressivity (38), more reactive, engaging and fidgeting behaviours (including head movements, laughing, smiling, general mouth movements, raised eyebrows, head touching and illustrative gestures), and fewer behaviours associated with disengagement after 6–8 months of deep brain stimulation (44, 47).

3 studies observed NVB changes whilst patients underwent courses of psychotherapy, and found increased positive emotional valence (41), increased expression entropy (45), and increased nonverbal synchrony (49) as treatment progressed.

### Effects of medication on NVB

In 12/20 studies, patients commenced medication or treatment at the same time of the baseline interview (30, 35–39, 41, 44–48). 2/20 studies included patients who were treatment naïve or medicated at the baseline interview (42, 50), and the remaining 6/20 studies did not specify if patients were already being treated prior to or commencing treatment at the time of the baseline interview (16, 34, 40, 43, 49, 51).

5 studies acknowledged and discussed the impact of medication effects on the study results. 1/5 studied the effect of medication on NVB in patients with depression, 1 included a sample of both patients with depression and patients with schizophrenia, and 3 explored the effect of medication on NVB in patients with schizophrenia (35, 42, 46, 48, 50).

In studies observing NVBs in patients with depression, Bouhuys et al. (1992) (35) mentioned that the pattern of behaviours at baseline did not significantly differ between medicated and unmedicated groups, but acknowledged that treatment conditions in the study were not controlled for which may have impacted findings.

Mackintosh et al. (1983) (46) studied a combined sample of patients with schizophrenia and patients with depression and found that there were no significant differences in the blink rate when patients were compared based on medication (patients were receiving one of three types of treatment: minor tranquilisers, tricyclic antidepressants, or major tranquilisers combined with antidepressants).

The studies exploring medication effects in schizophrenia described the impact on results in more depth. Meya and Renfordt (1986) (48) compared the effect of haloperidol and perazine in previously unmedicated patients with schizophrenia, and found that patients treated with haloperidol showed a tendency towards increased eye contact with the interviewer during the first 14 days of treatment compared to patients treated with perazine. The authors stated that more research is required to validate this finding and explore why this may have occurred.

Gaebel and Wölwer (2004) (42) conducted exploratory analysis of differential treatment effects of haloperidol and perazine in patients with schizophrenia, and found a significant medication effect regarding the facial repertoire and facial activity in the acute phase of schizophrenia, stating that an attenuation of facial expressivity between baseline and T1 was more pronounced in patients treated with haloperidol than perazine, and more pronounced in patients treated with perazine versus unmedicated control participants. When comparing unmedicated versus medicated patients at baseline, there was no significant difference in facial expressivity. The authors discussed that attenuated facial activity seen in the study is unlikely to be due to drug treatment and more likely to be explained by habituation to the interview, stating that the effect size was too small to be of clinical significance. Gaebel and Wölwer’s (2004) study did not align with the results observed by Meya and Renfordt (1986), signifying that the question of the influence of haloperidol and perazaine on alterations in NVB remains unanswered.

Further, Schneider et al. (1992) (50), included one group of patients (group 1) with schizophrenia who were unmedicated at the start of the study, and group 2 who commenced the study whilst continuing to take neuroleptic medication. The study found a significant reduction of facial action over time in group 1, and no significant change in group 2, despite there being no significant difference at baseline between groups in psychopathology scores. The authors also stated that more research is required to determine if reduced activity induced by neuroleptics is due to both motor and emotional sedation. The lack of consistent conclusions across all three studies represents a significant gap in the literature on the effect of medication on NVB in schizophrenia and the subsequent relationship to symptom scores and patient outcomes.

### Risk of bias assessment

Results from the risk of bias assessment using the CASP Cohort Study checklist (32) are presented in **Table 4**. Regarding areas of concern, just 9/20 studies clearly identified all confounding factors, 8/20 studies accurately measured the exposure (e.g. interview condition) and outcomes (clinical outcome and NVBs) to minimise bias, and only 5/20 studies had results that could be applied to the local population due to small sample sizes.

**Table 4:** Results from risk of bias assessment conducted using the CASP cohort study checklist.

Study	*1. Did the study address a clearly focused issue?*	*2. Was the cohort recruited in an acceptable way?*	*3. Was the exposure accurately measured to minimise bias?*	*4. Was the outcome accurately measured to minimise bias?*	*5a. Have the authors identified all important confounding factors?*	*5b. Have they taken account of the confounding factors in the design and/or analysis?*	*6a. Was the follow up of subjects complete enough?*	*6b. Was the follow up of subjects long enough?*	*9. Do you believe the results?*	*10. Can the results be applied to the local population?*
*Bhatia* et al.*, 2019 (*34)	Y	Y	N	N	Y	?	Y	Y	?	N
*Bouhuys & Albersnagel, 1992 (*35)	Y	?	?	?	?	Y	Y	Y	?	?
*Bouhuys* et al.*, 1986 (*36)	Y	?	?	?	?	Y	Y	Y	Y	Y
*Bouhuys* et al.*, 1987 (*37)	Y	?	?	?	?	Y	Y	Y	Y	Y
*Fiquer* et al.*, 2013 (*38)	Y	Y	Y	Y	Y	Y	Y	?	Y	Y
*Fiquer* et al.*, 2017 (*39)	Y	Y	?	Y	?	Y	?	Y	Y	?
*Fisch* et al.*, 1983 (*40)	Y	Y	?	Y	N	N	Y	?	?	?
*Fisher* et al.*, 2023 (*41)	Y	Y	Y	Y	Y	Y	Y	Y	Y	?
*Gaebel & Wölwer, 2004 (*42)	Y	?	Y	Y	?	Y	Y	Y	Y	Y
*Girard* et al.*, 2013 (*43)	Y	?	?	?	?	?	Y	?	Y	?
*Girard* et al.*, 2014 (*16)	Y	?	?	?	?	N	?	Y	?	?
*Jiang* et al.*, 2021 (*44)	Y	Y	?	?	?	?	Y	Y	Y	?
*Jones & Pansa, 1979 (*30)	Y	?	?	?	Y	?	?	?	?	?
*Liu* et al.*, 2022 (*45)	Y	Y	?	Y	Y	N	N	?	Y	?
*Mackintosh* et al.*, 1983 (*46)	Y	?	Y	Y	?	Y	?	Y	Y	?
*McCall* et al.*, 2020 (*47)	Y	Y	Y	?	Y	N	Y	Y	Y	?
*Meya & Renfordt, 1986 (*48)	Y	Y	Y	?	Y	Y	Y	Y	Y	?
*Paulick* et al.*, 2018 (*49)	Y	Y	Y	Y	Y	Y	Y	Y	Y	Y
*Schneider* et al*, 1992 (*50)	Y	?	Y	?	Y	N	Y	?	?	?
*Ulrich & Harms, 1985* (51)	Y	?	?	N	?	N	Y	?	?	?

I Question 11: Do the results of this study fit with other available evidence? Was excluded from the table due to heterogeneity of available evidence and no clear consensus. Questions 7 and 8 of the checklist are addressed in **Table 2**. ? indicates ‘Can’t tell’.

Strengths of the evidence base included that all studies addressed a clearly focused issue, 15/20 studies had a follow up of subjects that was sufficiently complete, and 13/20 studies followed up subjects for long enough.

## Discussion

To our knowledge, this is the first systematic review on how NVBs change over time as a response to treatment in patients with depression and schizophrenia. Methodological differences and a lack of studies including patients with schizophrenia made comparisons across studies and drawing conclusions challenging. Specific behavioural differences associated with improvement in depression symptoms that were found across multiple studies was increased mouth movements/smiling. No commonalities in NVB changes over time were observed for patients with schizophrenia. The degree of consistency across the literature was larger when our synthesis looked at broader behaviours associated with movement and expressivity and saw that such behaviours increased as clinical improvement occurred in patients with depression. In the majority of studies, patients commenced medication or treatment at the same time as they commenced involvement in the study, so these findings are perhaps most applicable to the earlier stages of treatment initiation following hospital admission or presentation to services, rather than monitoring long-term treatment responses in clinically stable patients.

Consistencies across intervention types suggests that broad categories of behaviour or movement and expressivity may also be less influenced by the means of analysis and by intervention – which better reflects the reality of how future remote-monitoring technologies could be used in clinical practice due to patient variation. In addition, the benefits of this approach are that it may be easier to validate such findings in a larger scale study using generalised automated analysis of behaviour rather than manual coding of specific AUs. A recent review of automated depression recognition highlighted that analysis of body expressions for the automated identification of depression is an important research avenue that should be explored in addition to research on facial expression analysis to identify depression (23).

### Evidence limitations

Sources of bias were largely related to the study design. Many studies did not consider confounding factors – e.g., if raters were blinded, if the same interviewer was used throughout, if the interviewer was familiar to the patient, which medications and dose were prescribed, and other patient demographics or comorbidities which may influence behaviours. The impact of cultural differences could not be extensively discussed due to limited reporting of patient demographics and limited acknowledgement of the impact of cultural factors within the included studies.

We also noted how the use of a variety of outcome measures when evaluating clinical improvement may account for the differences between studies. For example, across studies that used the HRSD to measure clinical outcomes we observed discrepancies regarding thresholds and questionnaire versions.

More is also to be understood about how NVBs change throughout the interview process. For example, reductions in NVB may occur due to habituation to the interview process (42). Several studies mitigated this by only recording NVBs during the first part of the interview (36, 38, 47). Still, studies that used this method differed in the length of the initial segment of the interview that was coded, ranging from 5–20 minutes.

Participants will also have increased familiarity with the interviewer throughout subsequent sessions which may impact NVB and introduces variation and uncertainty whereby patients within the same study with differing levels of familiarity with the interviewer are compared. Bhatia et al. (2019) acknowledged that one potential reason for not observing increased head movement synchrony between the patient and interviewer as clinical improvement occurred could be due to a lack of consistency in whether or not patients saw the same therapist during the 21 week trial – some patients had the same therapist, others may have changed therapist up to 4 times. This phenomenon has been recorded in the wider literature whereby the interviewer’s NVBs and nonverbal synchrony may influence patient improvement (18, 53).

Such sources of bias are implicit to using a clinical interview as the environment in which NVBs are measured. The benefits of assessing behaviours during a clinical interview are that it enables the observation of a larger range of behaviours that would occur during a social interaction which may not occur during an image viewing task or another means of assessing behaviour, however it is important to note that results from such studies may be influenced by the interpersonal relationship between the patient and the interviewer.

Areas of potential bias, such as expectation bias from the NVB raters if they are aware of the patient’s clinical status, can be overcome with the increasing use of automated means of NVB analysis. The use of automated analysis in future studies may also help to study the potential habituation to the interview by coding behaviours occurring across the entire interview, as it can be inferred that a reason for only analysing segments of interviews is due to the time burden associated with manual coding.

### Depression

General increases in movement and expressivity seen over time in patients with depression is in line with the Social Withdrawal hypothesis of depression, which suggests that patients with high levels of depression show less social or affiliative behaviours to maintain or increase interpersonal distance (16); a response that may have biological and evolutionary roots to protect the self from failure and/or harm (54–56). These observations could translate into a biomarker for assessing treatment response in depression.

There were studies outside the scope of this review which supported these findings. Two studies of NVBs in remitted patients with depression revealed that patients who went on to relapse displayed less active listening behaviours during the discharge interview compared to patients who remitted, and patients who showed less involvement behaviours at baseline had less favourable outcomes (57, 58).

Results which conflicted with this hypothesis were reported by Bouhuys and Albersnagel (1992) (35) and Fiquer et al. (2017) (39) who saw more speaking effort and restlessness in patients who did not improve. A potential explanation was that when patients with high speaking effort evoke excessive social support, they may be more prone to interpersonal distress and depression maintenance and persistence (39). Gaebel and Wölwer (42) (2004) found that patients had reduced expressivity as symptoms improved and that facial behaviours stabilised over a 3 month period. The authors stated that the reason for NVBs not changing as clinical improvement occurs could be due to habituation to the interview setting or a mismatch between the experience of emotion and displaying the emotion via facial expressions. Another explanation is offered by Fisher et al. (41) (2023) who found that emotional expressions follow a U shaped curve whereby positive emotions reduce as patients face challenging emotions in the middle of a course of therapy, but that the level of positive emotions at the end of treatment is higher than at the beginning.

One study outside of the scope of this review compared NVBs between patients with depression and a healthy control group. At baseline, the patient group showed higher levels of negative NVB (frowning, crying, head and lips down), and lower levels of positive NVB (eye contact and smiling). However, after eight weeks of treatment the patients with depression had no significant changes in their NVBs regardless of clinical improvement. The authors suggested that NVB may be independent of depression severity and may rather be a sign of depression (59).

We identified several noteworthy papers that investigated how NVB can be used to predict clinical change prospectively or retrospectively but could not be included in our review as NVB was only measured at one time point (57, 58, 60–62). Two papers studied behavioural predictors of amitriptyline response in depressed inpatients (60, 61). One showed that patients who did not respond to medication showed more body-focused movements, postural changes and reduced smiling at the baseline interview (61). Another found that non-responders demonstrated more assertive and affiliative behaviours (defined as a behaviour with an attractive function to draw the attention of the interviewer to the individual, and that express communicative efforts for social interaction) and had more engagement with the interviewer, compared to no significant behaviours associated with treatment responders (60). These findings from the wider literature contrast with results from this review in which clinical improvement in depression is associated with affiliative and interactive NVBs. This further exemplifies the need for larger scale studies and longer term follow ups that span the entire course of treatment.

### Schizophrenia

There were no consistently occurring behaviours identified across studies in patients with schizophrenia. Results were inconclusive from so few studies thus it cannot be determined if NVBs are a useful metric to measure treatment response in schizophrenia. Further research is needed to discern the impact of medication on NVBs and whether behaviour changes are a result of medication side effects or symptom improvement. There is a need to identify patients at risk of deterioration, and conversely, early identification of patients who may be improving, to guide treatment strategy. Treatment resistance is linked with a higher risk of clinical deterioration, hospitalisation, poor quality of life and impaired real-world functioning, therefore the early identification of patients who fail to respond to initial interventions can improve the treatment approach at an earlier phase and contribute to improving outcomes.

The paucity of research in this area is exemplified by the fact that no studies since 2004 have been conducted to further explore the role of NVBs in monitoring treatment response over time in schizophrenia. Specifically, the lack of studies on clinical deterioration could allude to potential reporting or publication bias, in that many of the NVB studies assessed in this review were conducted in parallel with an interventional clinical trial, and only studies which saw clinical improvement in patients (rather than deterioration) may have ultimately sought publication. In addition, it may be more challenging to recruit patients into long-term studies who could be more acutely unwell or at risk of deterioration.

NVBs could be a potential avenue on the search for reliable signals that allude to early treatment response, however the literature, in its current format, does not support this use.

There are two important factors which may have a strong influence over NVBs in patients with schizophrenia: the impact of negative symptoms and the effects of medication. Patients with higher levels of negative symptoms typically show reduced levels of NVB expression and a greater avoidance of social contact (63, 64). Avolition and blunted affect can impede social perception in patients with schizophrenia and ultimately reduce NVB expression (65). Furthermore, the presence of negative symptoms may be associated with a higher rate of spontaneous movement disorders in patients prior to any exposure to antipsychotic medication which may further affect NVB production (66). Movement disorders such as spontaneous dyskinesia or parkinsonism may therefore be related to the pathophysiology of schizophrenia in a proportion of patients rather than a side effect of antipsychotics, though medication may also alter the expression of NVBs (67, 68).

Known side effects of antipsychotic medication include reduced ability to move the eyes, facial activity, blink rate, and emotional bluntness (46, 50, 69, 70). Two studies included in the review discussed the potential impact of antipsychotic medication on facial expressions and reported conflicting results. One concluded that attenuated facial expressivity in patients with schizophrenia over time is unlikely to be due to medication effects, is unlikely to be clinically significant and is more likely to be attributable to habituation to the interview process (42). Conversely, Schneider et al. concluded that the reduction in facial expression is likely to be due to medication effects. Patients who were already medicated at baseline showed no change in their facial expression over time. Patients who were unmedicated at baseline but commenced medication during the study showed reduced facial expression over time, despite no significant difference in the psychopathology scores between groups. This finding raises questions about whether the observed reduction in movement is a result of high levels of agitation at baseline or due to side effects of antipsychotic medication and EPSEs (50). It is important to note the small sample sizes in either study (n=16 and n=34), exemplifying the importance of further research. A potential use of the further study of NVB in schizophrenia could be in trials comparing different antipsychotic regimes to determine the level of impact of the medication on NVBs and emotional blunting, and how alleviating this could support the expression of prosocial behaviours and social functioning, and facilitate recovery (17).

The study design may also influence the lack of consistent findings across patients with schizophrenia. Patients with different subtypes were included together in the analysis but considering different manifestations of schizophrenia and experiences of positive and negative symptoms, this may have not been appropriate. Compared to depression, the field of schizophrenia is far less researched and despite the implicit challenges outlined above, there is considerable scope in the evaluation of NVBs as predictors of clinical outcomes or change. This could be demonstrated in a large-scale, long-term study considering confounding factors (medication type and dosage, medical history, positive and negative symptoms) that have not been sufficiently accounted for in the existing literature to confirm or dispute historic findings. This is especially important considering that the last papers exploring NVBs in patients with schizophrenia were published 20 years ago so do not reflect recent treatment advancements.

### Strengths and limitations

To our knowledge, this is the first systematic review to examine NVB indicators of treatment response in adults with SMI. We performed a robust systematic search in line with PRISMA guidelines. Limitations of the review relate to the limited evidence and lack of large-scale studies, meaning the results must be interpreted with caution when generalising to the wider patient population. Additionally, requiring studies to record NVB at multiple time points or only during a clinical interview may have excluded other relevant papers that provide predictive insight into how NVBs relate to clinical improvement or deterioration.

Due to the heterogeneity of methods used to measure NVB’s, specific definitions used to define behaviours or groups of behaviours may vary across studies whereby the same behaviour is measured differently, which may be a limitation in our interpretation and analysis of behavioural results.

### Clinical practice considerations

Technologies that can automatically analyse facial expressions and emotions from video provide opportunities to incorporate real-time NVB analysis into routine video consultations or smartphone-based monitoring software (71), particularly given the increase in remotely delivered mental health care and the growing market for mental health apps (72). This integration of NVB analysis into routine mental health assessment and treatment processes could be beneficial to its broader adoption and consistent use, given that engagement with mental health apps is typically low and attrition is high, particularly if such technologies do not integrate seamlessly into mental health services (73). Having a behavioural biomarker that can be assessed in real time (such as NVBs being monitored during a routine consultation) may be preferable to the need for additional apps and technologies that collect data outside of clinical consultations (72). This may also reduce the burden of data collection and monitoring for both patients and healthcare professionals. This review concluded that there are several behavioural changes that could indicate clinical improvement in depression (head movements, quantity of movements, smiling). These are fairly simple, objective and easy-to-recognise behavioural markers that if validated further, could reasonably be tracked and monitored in real time during appointments.

Telemedicine and remote patient monitoring have led to statistically significant improvements in health outcomes, healthcare costs, patient satisfaction and accessibility across many disease areas (74). There is not yet a large evidence base on outcomes from the use of novel mental health technologies (75), and much more research is needed to address unknowns regarding the longer-term consequences and potential unintended consequences of digital monitoring interventions (72). Yet the fact that innovative monitoring technologies have been successfully integrated into care pathways in other chronic disease areas shows that enhancing mental health services with advanced digital solutions and novel behavioural biomarkers is a realistic possibility (73).

### Future recommendations

It is important to emphasise the potential challenges of assessing NVBs in psychiatric disorders. Firstly, the NVBs associated with a particular mental illness may vary across individuals within the same group, making it difficult to develop standardised assessments^18^. Additionally, the presence of mental, physical, and/or substance use comorbidities can further complicate the assessment of NVB (76–79). Similarly, treatment can affect the expression of NVBs, often not allowing to separate the effects of the illness from medication side effects. Therefore, future research should consider controlling for comorbidities, effects of medication and individual differences.

To improve the accuracy and reliability of NVBs as predictors for treatment response, future studies may benefit from standardising and controlling confounding variables and clinician and rater’s blindness to patient’s clinical or treatment status. Due to the sample sizes being small for most of the studies, future research would benefit from observing changes in NVBs on a larger scale of patients with a range of symptom severities and longer follow up periods, with strictly controlled treatment regimens and a control group where possible. We also noted a lack of studies on clinical deterioration. Designing future studies to focus on potential markers of early deterioration and relapse, rather than just clinical improvement, is an important area of further research and will further enable us to understand the utility of NVB biomarkers in either case.

Furthermore, if manual coding methods are used in future studies, having multiple raters, adequate training to improve inter-rater reliability and ensuring that interviews are consistent in timings and context may improve the reliability and validity of results. However, consideration should be given to the use of AI-based technology and automated methods to detect and analyse NVBs, reducing the potential of human rater bias (80) and enhancing the speed and efficiency of analysis. Incorporating AI-based technology could also help to enable remote monitoring and larger scale, cross-cultural studies taking place (81). At the same time, it is critically important to establish the reliability, validity and ethical considerations of AI-based technology and the collection of sensitive behavioural data (82, 83). Given the novelty of the field, data protection and ethical frameworks for the collection of NVB data and development of AI-based tools are currently lacking but essential for the sustainable widespread use of such technologies that preserve patient autonomy (72, 75).

## Conclusion

The current evidence for the utility of NVBs in monitoring responses to treatment in serious mental illness is limited, variable and mostly inconclusive. Indeed, the literature in its current format is insufficient to describe an observable behavioural profile of clinical improvement in response to treatment in SMI patients. Few behaviours may be associated with clinical improvement in depressed patients such as head movements, quantity of movements and smiling which could be further explored. There is a paucity of research in other populations such as in patients with schizophrenia or bipolar disorder. Future research would significantly benefit from large-scale, high-quality studies, supported by automated analysis to enable the potential of NVBs to act as reliable markers (and if possible as early indicators), for treatment response to be robustly assessed.

## Data Availability

The original contributions presented in the study are included in the article/**Supplementary Material**. Further inquiries can be directed to the corresponding author.
