# Supplementary material for: Non-verbal behaviours as predictors for treatment response in patients with depression or schizophrenia: a systematic review
Source: Front Psychiatry. 2025 Dec 17;16:1643042. doi: 10.3389/fpsyt.2025.1643042 (PMC12754059; doi:10.3389/fpsyt.2025.1643042)
Supplement: Supplementary file 2 [file DataSheet1.pdf]

			Study design						Population							Intervention			Measurement				Outcomes						Study quality					
Name of study	Author	Year	Type of study	Start/end date and duration of participation	Location of study	Study inclusion and exclusion criteria	Diagnosis	Subgroups	Number of participants (and how many were included in the final analysis), control/comparison groups	Average age	Age range	Gender of participants	Ethnicity of participants	How were patients recruited	Any other participant data if recorded (e.g. length since diagnosis)	Type of treatment given	Dosage/treatment regime	Length of intervention	Method of non-verbal behaviour measurement and unit of measurement (e.g. manual, automatic, and what aspect of the behaviours was recorded e.g. frequency or duration over how long)	Categories of non-verbal behaviours measured	Length of follow up period and how many follow ups	Nature of interview	Outcomes assessed (e.g. clinical change)	Method used to record clinical change/unit of measurement	Quantify clinical change	Statistical test used	Method of aggregation	Results with mean and standard deviation	Confounding factors	Other sources of bias	Methods used to address missing data	How the study was funded	Was there a conflict of interest statement?	Overall quality from CASP assessment (low, medium, high risk)
