# Supplementary material for: Non-verbal behaviours as predictors for treatment response in patients with depression or schizophrenia: a systematic review
Source: Front Psychiatry. 2025 Dec 17;16:1643042. doi: 10.3389/fpsyt.2025.1643042 (PMC12754059; doi:10.3389/fpsyt.2025.1643042)
Supplement: Supplementary file 3 [file Table2.docx]

Supplementary material: Full search strategy

| Combined with AND | | | | | |
| --- | --- | --- | --- | --- | --- |
| Combined with OR | non?verbal communication* | major depressive disorder | in?patient* | interview* | outcome |
|  | etholog* | Serious mental illness | out?patient* |  | prognos* |
|  | body language | schizophreni* | patient* |  | predict* |
|  | movement* | bipolar disorder | clinical |  | time |
|  | nonverbal | psychosis |  |  | follow* |
|  | eye contact | depressed |  |  | course |
|  | facial express* | depression |  |  | treatment |
|  | gesture* | psychotic |  |  | respon* |
|  | speech | schizoaffective |  |  | recover* |
|  | vocal | mania |  |  | change |
|  | voice* | manic |  |  | improv* |
|  | posture |  |  |  | relaps* |
|  | looking |  |  |  | treated |
|  | blink* |  |  |  | anti?depressant* |
|  | gaze |  |  |  | anti?psychotic* |
|  | non?verbal behavio?r* |  |  |  | mood stabiliser |
|  | facial action coding system |  |  |  | therapy |
|  |  |  |  |  | discharge* |
|  |  |  |  |  | factor* |
|  |  |  |  |  | indicat* |
|  |  |  |  |  | detect* |

**Table 2:** The full search strategy used for database searching.

**Boolean search string:**

(((non?verbal communication*) OR etholog* OR (body language) OR movement* OR nonverbal OR (eye contact) OR (facial express*) OR gesture* OR speech OR vocal) OR (voice* OR posture OR looking OR blink* OR gaze OR (non?verbal behavio?r*))) AND (((major depressive disorder) OR (Serious mental illness) OR schizophreni* OR (bipolar disorder) OR psychosis OR depressed OR depression OR psychotic OR schizoaffective OR mania) OR manic) AND (in?patient* OR out?patient* OR patient* OR clinical ) AND interview* AND ((outcome OR prognos* OR predict* OR time OR follow* OR course OR treatment OR respon* OR recover* OR change) OR (improv* OR relaps* OR treated OR anti?depressant* OR anti?psychotic* OR (mood stabiliser) OR therapy OR respon* OR discharge* OR factor*) OR (indicat* OR detect*))
